# Transfer RNA Fragments in Diseases of Sensory Organs

**DOI:** 10.3390/ijms27094142

**Published:** 2026-05-06

**Authors:** Nikita Gulati, Zhongyu Yang, Yan X. Lin, Hameed Sanusi, Bianca Gonda, Dylan C. McNally, Alaina Stellwag, Madison C. Holmes, Rabiba Chaudhary, Johannah Stevenson, Kelly Lepouski, Lanae Johnson-Kleinpeter, Sathyanarayanan Vaidhyanathan, Maria E. Solesio, Andrey Grigoriev

**Affiliations:** 1 Department of Biology, Rutgers University, Camden, NJ 08102, USA; ng884@scarletmail.rutgers.edu (N.G.); dm1765@scarletmail.rutgers.edu (D.C.M.); acs323@scarletmail.rutgers.edu (A.S.); mh1969@scarletmail.rutgers.edu (M.C.H.); rc1598@scarletmail.rutgers.edu (R.C.); kml401@scarletmail.rutgers.edu (K.L.); lcj46@scarletmail.rutgers.edu (L.J.-K.); sv646@scarletmail.rutgers.edu (S.V.); m.solesio@rutgers.edu (M.E.S.); 2 Center for Computational and Integrative Biology, Rutgers University, Camden, NJ 08102, USA; zy515@scarletmail.rutgers.edu (Z.Y.); yxl1@scarletmail.rutgers.edu (Y.X.L.); hos5@scarletmail.rutgers.edu (H.S.); bg711@scarletmail.rutgers.edu (B.G.); js2731@scarletmail.rutgers.edu (J.S.)

**Keywords:** tRNA-derived fragments, sensory organs, vision, touch, hearing, taste, smell, angiogenesis, inflammation, oxidative stress, biomarkers

## Abstract

Transfer RNA-derived fragments (tRFs) have been recently recognized for their multiple roles in gene expression, including modulation of translation, mRNA stability, and cellular signaling pathways. Sensory organs, such as the eyes, skin, and oral cavity, are continuously exposed to environmental stressors, including oxidative stress, ultraviolet radiation, microbial challenges, and mechanical stimuli, making them particularly susceptible to dysregulation of RNA-mediated processes. This review comprehensively summarizes current evidence on the role of tRFs in sensory organ physiology and pathology with a focus on their involvement in key processes, such as angiogenesis, inflammation, immune regulation, and fibrosis. tRFs have been shown to influence critical signaling pathways that are central to diseases such as retinal neovascularization, inflammatory skin conditions, wound healing, tissue remodeling, etc. Despite these advances, the field remains limited by a lack of experimentally validated tRF-target interactions, as most available data rely on computational predictions. The findings from the literature emphasize the need for rigorous functional validation in disease-relevant models of tRFs in biofluids, such as saliva and serum, to support their potential as minimally invasive biomarkers. Further translational studies are required to fully elucidate their biological roles and explore their potential in diagnostic and therapeutic applications.

## 1. Introduction

**Figure 1:**
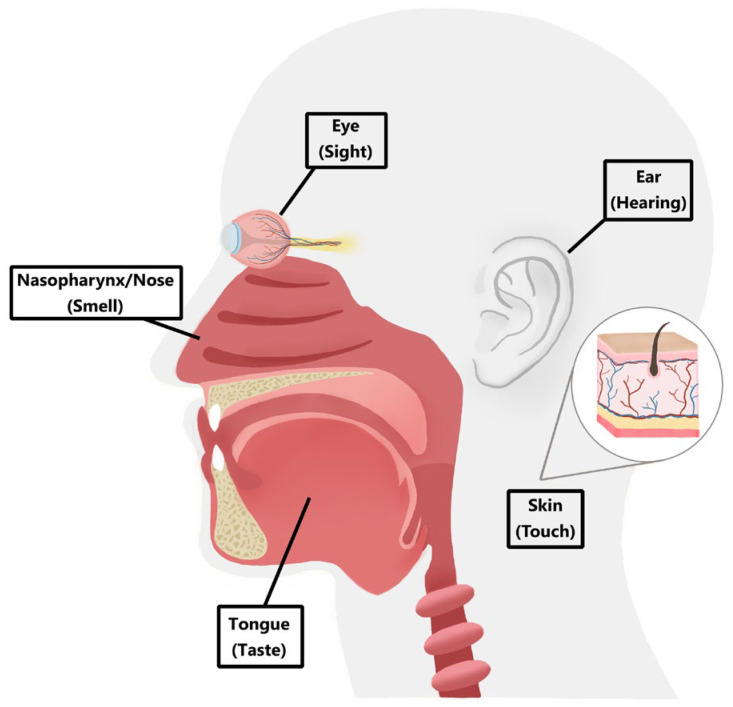
**Sensory systems organs.** Schematic overview of primary sensory organs, which serve as dynamic interfaces with the external environment and are therefore particularly susceptible to regulation by stress response-related tRFs.

Transfer RNA-derived fragments (tRFs) have recently emerged as a distinct and functional class of non-coding RNAs, produced from existing tRNA, and recognized to be not merely random degradation byproducts but rather novel regulatory molecules [1]. These fragments are generated through precise cleavage events mediated by various enzymes, including RNase Z, Dicer, and Angiogenin [2], particularly during stress conditions [3]. Our sensory organs serve as first-line defense detectors of systemic stress signals. Despite anatomical and regenerative capacity differences, all sensory organs, such as the eyes, skin, and oral cavity, experience high oxygen demand, exposure to reactive oxygen species (ROS) and mitochondrial stress, microbial challenges, and mechanical stimuli, making them metabolically vulnerable to regulation by genetic and epigenetic mechanisms. Because these sensory organs are continuously exposed to environmental stimuli, they provide an ideal system for studying interactions between regulatory RNAs and environmental factors (Figure 1). Understanding the fundamental functional role of tRFs in the broad range of functional regulatory processes, like vascular dysfunction and immune imbalance affecting various sensory organs, also highlights their evolutionarily conserved nature, influencing various integral cellular functions [4].

tRFs function via several mechanisms (see [5] for a detailed review) affecting gene expression. They are often considered similar to microRNAs (in most of the papers we review here) and act via Argonaute (AGO)-associated post-transcriptional regulation of target mRNAs [6]. Consistent with this view, in our earlier large-scale computational studies, we identified and placed in a database thousands of tRFs that target coding sequences and untranslated regions (UTR) of protein-coding genes [7,8,9]. Such targeting is based on the complementarity of tRF and target sequences, and was hypothesized to be the origin of the RNA interference, prior to the emergence of microRNA [10]. 

Through such targeting and/or interactions with specific RNA-binding proteins (or even directly with ribosomes [11]), tRFs modulate multiple angiogenic, inflammatory, metabolic, and viral replication pathways relevant to sensory organ function. Several fragments have been validated by luciferase reporter assays and RNA pull-down assays to regulate key mediators of signaling pathways involved in disease, including VEGF signaling, HIF-1α activation, and TGFβ-driven fibrosis [12,13]. tRFs have been reported to influence a variety of processes, including cell proliferation, migration, glycolytic metabolism, mitochondrial oxidative responses, and cytokine production, in a large number of disease conditions [14], of which cancer has received the most study [15]. In addition, tRFs regulate macrophage polarization, neutrophil activation, and interferon signaling, highlighting the role of innate and adaptive immunity [16]. They act as “dual modulators of pathogenesis”, either alleviating or aggravating disease progression. So, they may be promising as clinical therapeutic modulators [17].

Research on tRFs in the visual system has focused extensively on diabetes complications, ocular vascular disorders, and other common conditions like cataracts. The retina’s exceptional metabolic demand, with tightly regulated vascular networks and sensitivity to inflammatory imbalances, creates conditions in which rapid post-transcriptional control mechanisms are essential for cellular survival [18]. tRFs act as stress response regulators capable of modulating translation and metabolic pathways, influencing the progression of ocular vascular and neurodegenerative disorders that initiate vision-threatening diseases [19].

As the body’s largest organ and a primary sensory interface, the skin utilizes tRFs in response to environmental and immune challenges. tRFs are implicated in autoimmune conditions like Lupus and Psoriasis, influencing macrophage polarization and T-cell differentiation, as well as in wound healing and the response to UV radiation and photoaging [20]. 

Our sensory organs share a common pathogenetic foundation in immune dysregulation, where chronic inflammation drives endothelial dysfunction and cytokine imbalance. Emerging evidence links tRFs to the regulation of the oral microbiome and malignancies affecting the oral and nasal cavities. In the nasopharynx, distinct tRFs profiles have been identified in response to respiratory viruses, including respiratory syncytial virus (RSV) and SARS-CoV-2 [21], suggesting pathogen-specific activation of these fragments.

tRFs are abundant in biofluids, such as aqueous and vitreous humor and saliva (detectable in non-invasive samples); they remain stable in these biofluids. Accordingly, they can be used as accessible early disease-specific signature molecules, positioning them as valuable diagnostic and prognostic biomarkers [22]. This fact can be used to deliver tRFs mimics or their antisense oligos, potentially packaged in exosomes or similar vesicles [5]. 

However, most studies focus on one to two tRFs in a single disease and organ, so there is no unified integrated basis for the role of tRFs in sensory organ biology. Moreover, cross-study comparison is also a challenge as different naming systems are used (see [14] for a detailed description of such systems). Some of these systems involve using special software or websites that sometimes may go down for various reasons, including funding lapses. For example, the same tRF can be called tRF-3001a, 3′-tRF-LeuAAG, tRF-18-HR0VX6D2, LeuAAG-001-N-3p-68-85, or tDR-59:76-Leu-AAG-1-M6 by these systems. So, there is no universal solution to referencing tRFs at the moment, and this affects progress in the field. Ultimately, the tRF sequence is its best identifier (as identical sequences can come from different tRNA genes) and likely the single strongest determinant of its function. As in our earlier review [14], we circumvented this by putting all sequences in Table A1 in Appendix A and naming them by a reference number. So, a tRF found in reference XX is named TXXa in the text below, and for multiple tRFs from the same publication, we use the notation TXXa, TXXb, and so on.

In this paper, we reviewed evidence from studies of different depths, from pure predictions (type I) and target-binding reporter data (type II) to functional validation (type III), following our earlier classification [14]. Upon sequencing tRFs, type I studies (following the assumptions of microRNA-like action of tRFs) typically utilize only computational tools, like TargetScan [23], to map putative tRF targets to equally putative biological processes via Gene Ontology (GO) terms and similar pathway analyses. Type II papers test interactions of tRFs and their targets with luciferase reporter assays and sometimes analyze the expression of the targets with qRT-PCR or Western blots. Type III studies use further functional validation of tRF role involving cellular functional assays, metabolic analysis, and in vivo disease models, providing multi-level evidence for regulatory activity of tRFs. We indicate these types in our review to synthesize the current understanding of tRFs within the context of sensory organs, where they have been implicated in a wide range of physiological and pathological states.

## 2. tRFs in Eye-Related Conditions

The potential roles of tRFs have been investigated in multiple diabetic ocular complications, ocular vascular disorders, and other eye-related diseases, detecting links with various ocular molecular pathways regulating vascular dysfunction, inflammation, and neovascularization. These tRFs can be reliably detected in ocular biofluids, such as aqueous and vitreous humor, which holds a high diagnostic and prognostic value in vascular pathologies. Also, therapeutic modulation can make the tRFs a potential adjunct in ocular drug therapy. Numerous in vitro and in vivo studies, including a range of viability and functional assays (Table 1), have demonstrated the involvement of tRFs in the pathogenesis and progression of various ocular disorders.

### 2.1. Diabetic Retinopathy 

Various tRFs exhibit different abundances in patients with diabetic retinopathy (DR) and could serve as biomarkers or therapeutic targets [24,25]. Type I predictions have linked these tRFs to GO terms related to cellular macromolecule metabolism, AMPK signaling, and ubiquitin-mediated proteolysis, potentially relevant for the pathogenesis and treatment of DR. 

Several focused studies suggested specific tRF targets in DR. In vitro cell function assays and in vivo vascular assays showed that T26a lessened DR-related effects’ complications [26], while luciferase and RNA pull-down assays confirmed its direct binding to mRNA of DVL2, a member of the dishevelled family and a key regulator of the Wnt/β-catenin signaling pathway. Overexpression of DVL2 reversed its protective effects, indicating that T26a alleviates DR symptoms by reducing DVL2 expression. 

T27a is increased in high-glucose conditions, and it has been shown to worsen DR-related angiogenesis by enhancing cell proliferation in vitro [27]. Binding to the 3′-UTR of and repressing SIRT1 translation, it induces HIF1A activation and leads to excessive retinal neovascularization, which is a key feature of DR. Decreasing the abundance of T27a in DR mice improved DR symptoms, with a notable increase in SIRT1 and a decrease in HIF-1α protein levels.

Similarly, T28a was significantly increased in the retinas of diabetic mice, worsening DR-related angiogenesis [28]. In vitro, its direct binding to and repression of CD73 decreased adenosine production, which leads to retinal vascular dysfunction, indicating that this tRF aggravated DR complications by removing adenosine’s protective effects.

Another tRF, T19a, was upregulated in both in vivo and in vitro DR models, where it aggravated retinal vascular dysfunction [19]. Direct binding led to overexpression of TRIB3, a regulator of cell proliferation and STAT3 signaling. By increasing TRIB3 expression and consequently dysregulating cell proliferation, this tRF was proposed to exacerbate DR symptoms.

### 2.2. Neurovascular Dysfunction

Neurovascular dysfunction is a major part of diabetes-related eye disease, and several tRFs have been linked to these complications. T29a and T30a were strongly induced by diabetic stress in cells, diabetic mice, and patients with diabetic retinopathy [29,30]. 

Luciferase assays confirmed the direct binding of T29a to GSK3B, driving both vascular and neuronal dysfunction in vitro and in vivo. Lowered abundance of T29a reduced abnormal blood vessel growth, inflammation, and overall retinal nerve cell damage [29]. 

A significant elevation of T30a was found in diabetic retinas, Müller cells, and diabetic patient samples. qRT-PCR assays suggested this tRF likely targets CYP2E1. Metabolomic analysis and ELISA assays demonstrated increased arachidonic acid metabolism and inflammation, while Evans Blue assays confirmed enhanced retinal vascular leakage, driving diabetic retinal neurovascular dysfunction in vitro and in vivo. Reducing T30a improves vascular stability, limits neurodegeneration, and increases visual discrimination acuity [30].

### 2.3. Myopia

Myopia progression is driven by scleral hypoxia, aggravated by choroidal vascular dysfunctions. In a study using C57BL/6 J mice, FDM followed by the CNV model was induced, where T31a reduced vitreous chamber depth, elongation, and axial length [31]. It binds to the 3′-UTR of METTL3, as confirmed by luciferase assay, blocks METTL3-mediated m6A methylation of Axin1 or Arid1b mRNA, and releases their inhibitory effect on Wnt signaling. m6A-bound YTHDF2 reduces Axin1 and Arid1b levels. In vitro, METTL3 overexpression enhances RF/6A cell proliferation, migration, and tube formation, whereas in vivo, it restores collagen production (by increasing COL1α), inhibits myofibroblast trans differentiation (by decreasing α-SMA and vinculin), and slows myopia progression. Clinically, METTL3 abundance is substantially lower in the aqueous humor of patients with myopic CNV, highlighting its deficit in driving the disease.

### 2.4. Age-Related Macular Degeneration

Age-related macular degeneration (AMD) is a major cause of blindness in elderly people, driven by choroidal neovascularization, which interrupts the blood–eye barrier. 

T18a is identified as a key promoter of hypoxia-triggered pathological angiogenesis in AMD. In luciferase assays, it targets VASH1, a modulator of VEGFR2 trafficking. In vivo, T18a downregulation promotes endothelial senescence via vimentin and inflammatory factor expression, reduction in laser-induced CNV lesion size, vascular leakage, and tissue hypoxia, whereas in vitro, it enhances endothelial cell migration, proliferation, tube formation, and choroidal sprouting. It reduced the CNV area by 50%, an effect comparable to that of bevacizumab (an anti-VEGF drug). ERG and OCT confirm that tRF does not affect total retinal layer thickness, indicating that it may be a potentially safe therapeutic strategy for neovascular AMD [18].

Long-term anti-VEGF therapy leads to resistance and subretinal fibrosis, highlighting the need for novel anti-angiogenic therapies. T13a, targeting VEGFA and TGF-β1, is a potential double controller of angiogenesis and inflammation for vision-related vascular lesions [13], since its abundance is markedly reduced in diabetic retinas, HRVEC, and RPE/choroids of the CNV mouse model. The delivery of this tRF via extracellular vesicles (EVs) exhibits a strong synergistic anti-angiogenic and anti-inflammatory effect, without detectable retinal structural abnormalities or apoptotic retinal cells gain, proving its stability but not short-term toxicity [13].

Several studies explored tRF’s role in angiogenesis and inflammation [32]. Various tRFs, such as T32a-T32f, were supported by qRT-PCR as upregulated and downregulated. Using type I predictions, most of them indicated GO term enrichment in endothelial proliferation, metabolic reprogramming, and enrichment in pathways involving VEGF and HIF1A genes [32], without validation experiments.

### 2.5. Ocular Angiogenesis

Ocular angiogenesis governs many retinal diseases, and OIR models are frequently used to address its status. T33a is upregulated in OIR, CNV, and HUVEC models. In vitro, its antisense oligo in HUVEC induces apoptosis and decreases cell proliferation, migration, and tube formation. In vivo, this oligo inhibits neovascular tufts formation in OIR and reduces CNV lesion size with efficacy comparable to aflibercept (anti-VEGF drug). Hence, blocking this tRF normalizes endothelial metabolism by suppressing aberrant neovascularization, offering a possible therapeutic alternative for ocular angiogenesis disorders [33].

A significant upregulation of T34a and T34b and downregulation of T34c and T34d was reported in the OIR model as mainly impacting targets enriched in (as suggested by type I predictions) GO terms related to immune responses and vascular development [34].

T12a is significantly downregulated in various ocular angiogenesis models, including neonatal OIR and CNV, as well as in the context of abnormal blood vessel growth, proliferative retinopathy, and AMD [12]. High levels of T12a reduce pathological vessels in vivo and inhibit endothelial cell migration, sprouting, and formation of the tip-cell in vitro. The tRF regulates endothelial sprouting by modulating angiogenesis by targeting RBPJ and MAML1, members of a METTL3 pathway. In a potential connection, mouse models of OIR and CNV show significantly decreased abundance of T35a, which enhances Notch signaling by suppressing METTL3-mediated m6A modifications that degrade RBPJ and MAML1 mRNAs [35]. 

### 2.6. Cataract

Increased oxidative stress is a known driver of age-related cataract. Increased oxidative stress is closely related to calcium dyshomeostasis. Type I predictions identified several tRFs with GO terms of predicted targets potentially linked to the calcium signaling pathway, which is critical for maintaining lens transparency [36]. Another study also briefly discussed tRFs’ functions in diabetic cataracts [37], and type I predictions of predicted target genes of T37a-T37e showed enrichment of GO terms involving the FoxO signaling pathway. 

### 2.7. Pterygium

Pterygium is characterized by fibrovascular overgrowth and recurrence. Functional assays revealed that T38a is a potent promoter of fibroblast proliferation and migration, hallmarks of pterygium growth and recurrence. Type I predictions identified its target gene names enriched with GO terms mentioning pathways of focal adhesion, TGF-β signaling, PI3K-Akt signaling, and ECM–receptor interaction [38].

### 2.8. Lymphangiogenesis

Lymphangiogenesis, a process crucial for maintaining tissue fluid balance, immune function, and lipid absorption, has a significant influence on ocular function, particularly through corneal lymphangiogenesis [39]. Corneal models demonstrated that T39a is consistently downregulated in inflammatory conditions in lymphatic endothelial cells (LECs). Restoring or overexpressing T39a suppressed LEC proliferation, migration, and tube formation and reduced lymphangiogenesis in vivo via inhibition of the glycolytic enzyme PKM2. Clinical samples from keratitis patients showed reduced T39a and elevated PKM2, confirming its pathological relevance [39].

### 2.9. Retinal Degeneration

A study demonstrated that interferon (IFN)-γ stimulation enhances the protective effects of mesenchymal stem cell (MSC)-derived EVs in a model of retinal degeneration by altering tRFs with targets associated with inflammatory and immune-related pathways [40]. Similarly, infants with treatment-requiring retinopathy of prematurity [41] showed dysregulation of tRFs in peripheral blood mononuclear cells. Type I predictions listed targets with GO terms enriched in pathways related to immune response, cellular metabolism, transcriptional regulation, Th17 cell differentiation, and FoxO signaling. 

## 3. tRFs in Skin-Related Conditions

**Figure 2:**
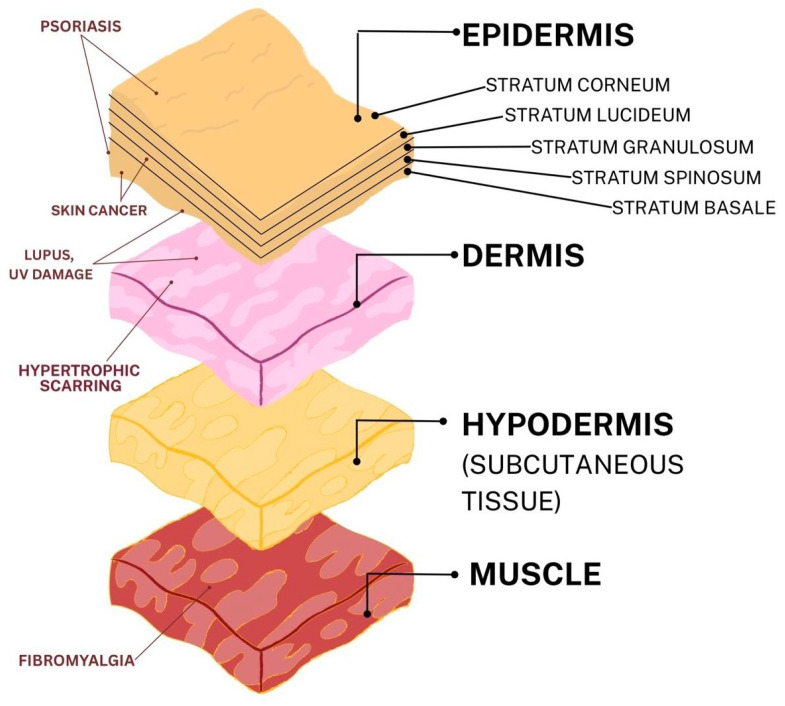
**Structural organization of the skin in the context of associated pathological conditions.** Schematic illustration of epidermal, dermal, and subcutaneous layers of skin, representing their structural complexity and susceptibility to pathological conditions. These layers represent a dynamic interface to various stressors, highlighting their potential as environments for the action of stress-related tRFs.

Skin is the largest organ in the human body and is typically seen as the sensory conduit of pain and/or touch. It also has a very large number of other prominent roles (Table 2) and a very complex structure. tRFs have been studied in pathologies related to multiple skin layers (Figure 2) and the roles they play. 

We have previously reviewed tRF roles in broader autoimmune, immune, and inflammatory processes [14], and many of those were somewhat relevant in the context of skin. Here, we focus specifically on the effects of tRFs in skin-related diseases and review recent efforts that have sought to decipher the roles tRFs may have across the various skin functions. 

### 3.1. Keloids, Wound Healing, and Fibromyalgia

In a lipopolysaccharide-stimulated macrophage cell model, T42a suppressed inflammatory signaling by targeting Cul4a, a cell-cycle regulator. Experiments show that this fragment attenuated NF-κB activation, a pathway that is centrally involved in keratinocyte activation, cytokine production, and chronic skin inflammation [42,43]. Similarly, a mimic of another tRF promoted M2 macrophage polarization in an undifferentiated macrophage cell line and mediated programming in the inflammatory environment of keloid tissue. As M2 macrophages are associated with increased proliferation, this fragment may contribute to the fibroproliferative response characteristic of keloids [44]. 

The influence of tRFs on the skin microenvironment is not limited to macrophages. In patient samples from diabetic foot-ulcer skin, transfection of the mimic T45a exacerbated high-glucose-induced endothelial dysfunction in human umbilical vein endothelial cells. This dysfunction inhibited many processes necessary for efficient wound healing [45]. Likewise, another study showed that T46a, T46b, and T46c were present at decreased levels in diabetic foot ulcer tissue compared to healthy skin. However, following hyperbaric oxygen treatment, these fragment levels markedly increased. Type I predictions suggested that Wnt signaling and cell growth-related genes and pathways might be affected by these three tRFs [46]. 

tRFs have also been found to play a role in pain and touch sensing. Fibromyalgia, a condition associated with inflammatory dysregulation, has widespread chronic pain as its hallmark symptom. T47a and T47b were shown to clinically correlate with skin denervation and small fiber pathology severity in the keratinocytes of fibromyalgia patients. Type I predictions of targets of differentially abundant tRFs were enriched for GO terms mentioning pathways related to cell adhesion, migration, and growth [47]. Finally, separate studies have shown that tRFs may also play a role in the immune response in animal models. For instance, in adult sheep, type I predictions and gene regulatory network construction hinted that tRFs may have an impact on the innate and adaptive immune pathways in juvenile vs. adult sheep skin [48].

### 3.2. Lupus

Systemic Lupus Erythematosus (SLE) is the most common form of lupus, resulting in a range of pathological issues [49]. It is caused by environmental and genetic factors that collectively interact to trigger an immune response consisting of the overproduction of pathogenic autoantibodies by B cells and cytokine dysregulation [50]. tRFs have been proposed to serve as a mediator in the immune response, as illustrated in the studies reviewed below.

Lupus autoantigen (La) is an RNA-binding protein involved in the RNAi pathway. La prevents loading of tRFs into AGO, binding them via two RNA recognition motifs in La. In La KD, the abundance of let-7 miRNA family members decreased, while Pro-, Ile-, and Gln-derived tRFs began to peak, and a reduction in interactions between Dicer and pre-tRNAs was present [51].

Macrophage polarization influences the balance of M1 to M2 macrophages, which plays a significant role in the inflammatory and proliferative processes of autoimmune diseases, such as SLE [52], similar to its role in keloids described above. Pro-inflammatory M1 macrophages are upregulated in SLE, and MSC-dependent therapies have shown efficacy in SLE models. MSC-derived EVs inhibited M1 while promoting M2 (anti-inflammatory) features in a macrophage cell line. T16a levels increased, and they could contribute to this immunomodulatory effect. In SLE patient samples, T16a levels were also significantly decreased, suggesting a disease-associated deficiency. Type I predictions suggested this tRF (a possible functional cargo of MSC EVs) targets enriched in GO terms on reprogramming macrophages toward a protective phenotype and represents a potential therapeutic mechanism for SLE [16].

In T cells, oxidative phosphorylation (OXPHOS) promotes the proliferation and upregulation of T cell genes [53]. This process is critical as overactive T cells in the autoimmune response in SLE contribute to disease progression [54]. Among hundreds of differentially regulated tRFs, T55a was elevated in SLE, correlating with active disease and serum IFN-α level. High levels of the tRF increased the oxygen consumption rate, ATP production, mitochondrial membrane potential, and ROS generation, while KD diminished IFN-α–induced OXPHOS. The authors described T55a as a metabolic modulator downstream of IFN-α signaling that promotes OXPHOS activation in lupus CD4^+^ T cells [55].

Neutrophil extracellular traps (NETs) are networks of extracellular strings of DNA that bind to pathogenic microbes, slowing disease progression in healthy individuals [56]. The authors show that immune complexes in SLE activate platelets via FcγRIIA, leading to the release of platelet-derived EVs enriched with T57a. These EVs are taken up by neutrophils, triggering ERK/p38 MAPK signaling, phosphorylation of p47phox, and increased ROS production, where T57a directly binds and activates Toll-like receptor 8, ultimately resulting in increased NET formation. In a KD experiment, T57a inhibitors effectively suppress NET formation and cytokine release, suggesting this axis as a promising therapeutic target in SLE, as it serves as a primary mechanism of pathological progression of the disease [57]. 

### 3.3. Psoriasis

Psoriasis is primarily a dendritic and T-cell mediated skin disease involving the dysregulation of neutrophils, keratinocytes, vascular endothelial cells, and antigen-presenting cells [58]. tRFs have been proposed to play a role in the regulation of psoriatic pathways and in overall disease progression.

For example, T20a serves a protective role in the pathological progression of psoriasis through a variety of different mechanisms, potentially including binding to the SERPINE1 3′UTR. Type I predictions of a differentially abundant tRF T20a in skin lesions of psoriasis patients indicated GO term enrichment of VEGF. Keratinocyte differentiation-associated proteins, specifically KRT1, KRT14, LOR, and IVL, were shown to change with increased T20a levels. This suggested the tRFs’ protective role and influence in angiogenesis could be exerted not only via suppression of SERPINE1 and HIF1A mRNAs but also through keratinocyte differentiation [20].

A study on rheumatoid and psoriatic arthritis solidified the significance of tRFs in inflammatory arthritis. Decreased levels of T59a, T59b, and T59c in the serum of rheumatoid arthritis patients and increased levels of T59b and T59c in psoriatic arthritis patients were identified. The authors also verified a similar decrease in T59b and T59c in an inflammatory arthritis mouse model. Since these two similar diseases have different circulating tRF profiles, these fragments may have diagnostic utility; however, further validation is needed [59]. 

### 3.4. Atopic Dermatitis

Atopic dermatitis is a common chronic, inflammatory dermatological condition resulting from a combination of immune, genetic, and environmental factors. Its precise pathogenesis is unknown. The roles of tRFs in pediatric atopic dermatitis (AD) were explored by small RNA sequencing of plasma from children with AD and healthy controls, identifying 31 differentially abundant tRFs. Type I analyses revealed that these fragments may not have reliably predictable targets. However, two tRFs were validated via RT-qPCR, and T60a was robust in predicting diagnosis but not the severity of the disease [60]. 

### 3.5. Photoaging and UV Damage

Using human dermal fibroblasts irradiated with repetitive UVA radiation as a photoaging model [61], type I prediction linked the putative targets of upregulated T61a, T61b, and T61c to the GO terms, mentioning the status of cell cycle and replication, oxidative stress, inflammation, and tissue damage. Downregulated tRFs, T61d, T61e, and T61f were linked to predicted target groups with GO terms, including interactions with viral proteins via cytokine receptors, complement cascades, and Th1 and Th2 cell differentiation [61].

Additionally, T62a was shown to induce photoaging by promoting cellular senescence and downregulating NUP98 in UVB-exposed mice. UVB exposure leads to activation of the JNK pathway, which mediates apoptosis. Dual luciferase assay, mimic transfection, and in vivo experiments demonstrated that inhibiting T62a reduces senescence-related gene expression and increases NUP98, highlighting its potential as a target for therapies against skin aging [62]. A complementary study of UVB-exposed mice showed that T63a targets TRPV3 and activates the PI3K/AKT pathway, ultimately leading to aging phenotypes [63]. A separate study used type I predictions of T64a targets with terms mentioning Wnt signaling by targeting Rac1, a regulator of DNA damage and keratinocyte apoptosis, in UVB-treated mice [64].

tRFs and other small non-coding RNAs are emerging as important regulators in skin cancer, namely in melanoma, where oxidative stress and UV-induced tRNA fragmentation may increase mutagenesis [43]. Additionally, comparative studies in animal models provide further evidence of tRF roles in skin cancer. Decreased levels of T65a were conserved across canine oral melanoma tissue and human oral and cutaneous melanoma cell lines [65]. Similarly, in a melanoma cell line, UVB radiation led to increased T66a. Mimic transfection of this tRF and its inhibitor in melanoma cells resulted in decreased CCDC88A expression, which decreased PI3K/Akt signaling, and induced upregulation of pigmentation genes to promote melanogenesis, placing T66a as a potential novel regulator of melanogenesis [66].

### 3.6. Hypertrophic Scarring

Hypertrophic scars, driven by excessive fibroblast proliferation and extracellular matrix accumulation, compromise skin function and appearance. Various differentially expressed tRFs in human hypertrophic scar fibroblasts have been explored, such as T67a, T67b, and T67c. Type I predictions suggested dysregulated tRFs targets with GO terms enriched mainly in cell adhesion, protein binding, angiogenesis, and actin binding, as well as altered pathways involved in Ras, Rap1, and cGMP-PKG signaling. These analyses also predicted T67c targets COL1A1, which would lead to abundant collagen and SMAD2 and TGFBR1 (TGF-β signaling), involved in fibroblast activation, inducing the epithelial–mesenchymal transition. These fibrosis-associated tRFs are potential drivers of scar formation and could be used to develop new treatment strategies [67].

### 3.7. Vascular Stenosis

tRFs deliver a crucial contribution to vascular remodeling mechanisms. In a rat carotid balloon injury model, T68a was significantly upregulated. It promotes neointimal hyperplasia by binding to 3′UTR and suppressing fibromodulin [68], which enhances TGF-β1/Smad3 signaling. It also increased cell proliferation and migration in vitro, whereas its anti-sense decreases both the neointimal area and the arterial intima/media area ratio by almost 60% in vivo [68]. Thus, post-injury inhibition of this tRF may prevent vascular stenosis, facilitating healing. 

## 4. tRFs in Oral, Nasopharyngeal, and Ear-Related Conditions

A significant involvement of tRFs has also been explored in various other sensory organs encompassing the oral cavity, nose, ear, and multiple associated body structures, as well as defense mechanisms against the viromes/microbiomes colonizing and affecting them (Table 3).

### 4.1. Oral Conditions/Diseases

#### 4.1.1. Microbiome Modulation

Two human tRFs, T69,70a and T69,70b, were studied to determine their role in modulating immune response to oral bacterial infections. These tRFs contributed to the growth inhibition of *Fusobacterium nucleatum*, a bacterium involved in dental plaque formation and periodontitis [69]. To further establish the role of these tRFs, a follow-up work focused on repurposing human tRFs as anti-microbial agents against *F. nucleatum*. The two tRF mimics were modified with 2′-O-methylation and a phosphorothioate bond, and they showed inhibition of the growth of *F. nucleatum* with improved stability over their naturally occurring counterparts, suggesting a possible role for tRFs as antimicrobials [70].

#### 4.1.2. Oral Squamous Cell Carcinoma

Head and neck oral squamous cell carcinoma (HNSCC) studies have linked the presence of small tRFs to the development and progression of cancer. T71a, T71b, and T71c were significantly associated with high survival rates in patients [71]. Another study identified 22 tRFs (and other small RNAs) whose abundance was significantly different in HNSCC and healthy individuals. Type I predictions and statistical analyses suggest WEE1 and FBXO31 as tRF targets. The expression of these genes, involved in the suppression of cell proliferation, was significantly reduced in tumor tissue in comparison to healthy tissue [72]. 

A computational analysis identified some significantly abundant tRFs and sought their anticorrelation with the expression of several tumor suppressor genes and oncogenes, as well as potential complementary binding sites on their mRNAs. The resulting model was a poor predictor of the disease state, as the authors noted the accuracy of their classifier was 57% when used in validating an external cohort [73]. However, this outcome may be related to the assumptions driving the study, e.g., research has shown that some TCGA-based tRF profiles did not match the findings of studies focusing on specific tRFs in specific cancers [74].

#### 4.1.3. Laryngeal Carcinoma

In laryngeal squamous cell carcinoma (LSCC), T75a was found to be highly downregulated, especially in advanced stages, where lymph node metastasis is present. The study postulates that this tRF inhibits tumor growth in LSCC tissues by suppressing cell proliferation, tumor migration, and invasion with PIK3CD as a potential target, confirmed by a luciferase assay [75]. While laryngeal cancer affects the larynx, hypopharyngeal cancer starts in the lower part of the throat, right behind the larynx. In hypopharyngeal cancer patients, T76a was found to be notably increased, implicating a possible role as a biomarker [76].

#### 4.1.4. Oral Melanoma

Investigations of oral melanoma in dogs have identified three tRFs that are potentially involved in the progression of the disease. T65b, T77a, and T65a levels were reduced in oral melanoma samples, with T65b specifically showing a reduction in canine melanoma samples and cell lines [65]. A later study by the same authors reported EV cargo tRFs to have a significantly increased abundance in oral melanoma cell lines, compared to controls [77]. These results confirm that tRFs may play a role in oral melanoma.

#### 4.1.5. Esophageal Carcinoma

T78a is significantly increased in esophageal carcinoma (ESCC) patients, compared with healthy samples. This observation was followed up by a clinical study that identified a bi-signature salivary small non-coding RNAs consisting of T78a and an uncharacterized small RNA in EVs. This prediction approach found that ESCC patients with a high Risk Score Prognosis (RSP) had shorter overall survival and progression-free survival than those with low RSP. Interestingly, patients with high RSP had improved overall survival when introduced to adjuvant therapy, while in patients with low RSP levels, survival did not change. Thus, T78a may be an effective biomarker and prediction tool that can determine if a patient with ESCC could benefit from additional cancer therapy [78].

#### 4.1.6. Non-Syndromic Cleft Palate

T79a and T79b were found to be consistently and significantly elevated in non-syndromic cleft palate tissues relative to healthy controls [79]. These dysregulated tRFs may perturb key developmental pathways by modulating the expression of critical regulatory genes, including BMP6, CUL1, and SPR. These genes exhibit reduced expression in affected tissues at both the transcript and protein levels, which may disrupt physiological palatal formation and offer mechanistic clues for cleft palate pathogenesis [79].

### 4.2. Nasopharyngeal Diseases 

#### 4.2.1. Nasopharyngeal Carcinoma

Recent studies suggest that dysregulated tRFs play an active role in upper respiratory tract diseases. In primary nasopharyngeal carcinoma (NPC), high-throughput tRF sequencing has revealed multiple differentially expressed tRFs. T80a, T80b, and T80c are found to be enriched in pathways central to tumor progression and immune modulation, highlighting their potential in biomarker development for NPC detection [80]. 

Another study established the role of T81a in promoting the development of NPC. Evidence suggests that the upregulation of this tRF level in NPC cells is significantly associated with cancer activity, such as the epithelial–mesenchymal transition, a key process in tumor progression, and the suppression of apoptosis. In vivo experiments using mouse models validated the ability of T81a to promote tumor growth. These effects were mediated by the modulation of EPHB2 gene expression [81].

#### 4.2.2. Infection by Respiratory Syncytial Virus (RSV)

Beyond malignancy, airway infection has been shown to trigger the highly selective induction of tRFs, among which T82,83a functions as a trans-silencing small RNA. Inhibition of this tRF reduces RSV-mediated induction of IL-8, RANTES, and IFN-β, positioning it as a potential therapeutic target in viral bronchiolitis [82]. More recent evidence suggests that during infection, RSV induces ALKBH1-mediated demethylation of m1A57 on tRNA-GluCTC, allowing its subsequent cleavage to produce T82,83a. Inhibition of ALKBH1 or disruption of T82,83a biogenesis markedly reduces viral protein production and replication [83].

Based on type I predictions and high-throughput sequencing of RNAs associated with biotinylated tRF (HITS-Abt), APOER2 was named a new anti-RSV protein, with its 3′-untranslated region being a target site for T84a. APOER2 acts as an antiviral protein by interacting with and sequestering the viral P protein to prevent the formation of a viral polymerase complex. To confirm the role of this tRF, APOER2 mRNAs were found to interact with its biotinylated form using a luciferase reporter assay. After tRF mimic transfection, APOER2 expression was significantly decreased upon RSV infection, while antisense oligonucleotide reversed this decrease [84].

A proteomics study identified that poly(A)-binding protein cytoplasmic 1 (PABPC1) may be interacting with RSV-induced tRF T85a. This was confirmed by immunoprecipitation methods, which detected PABPC1 in a complex with this tRF [85]. Suppression of PABPC1 via transfection of siRNA led to increased replication of the RSV(-) genome, while the knockdown of PABPC1 reduced cytokine production. However, the suppression of PABPC1 did not impact viral gene transcription but led to a decrease in viral load. Taken together, this study provides the first observation suggesting the involvement of tRFs in regulating viral infection via direct complexing with proteins [85].

SARS-CoV-2 infection significantly increased levels of several other tRFs in the nasopharynx [21,86], demonstrating that respiratory viral infections activate distinct, pathogen-specific tRF programs, which broadly rewire the nasopharyngeal environment in response to different viruses.

#### 4.2.3. Asthma

Given that tRFs circulate throughout the respiratory tract and reflect epithelial stress, their dysregulation in severe asthma provides a framework for understanding their roles in nasopharyngeal diseases. T87a and T87b are increased in the plasma and sputum of patients with severe asthma, alongside elevated levels of angiogenin in the inflamed respiratory microenvironment [87]. Inflammatory and pollutant stimuli further enhance angiogenin activity and tRF production, linking their dysregulation to respiratory immune stress. Importantly, altered tRF levels correlate with disrupted glucocorticoid receptor signaling, suggesting a role in the molecular mechanisms underlying corticosteroid insensitivity.

### 4.3. Ear Infection by Candida auris

*C. auris* infection can cause severe damage to the bloodstream, wounds, ears, and respiratory system, and can be fatal. It is highly resistant to antifungal drugs, such as the recommended treatment (caspofungin). Treatment with caspofungin increased production of EVs and tRFs transported by these EVs, T88a and T88b. Further investigation into these tRFs can identify their involvement in the mode of action of caspofungin while treating these infections [88].

## 5. Synthesizing the Information

Our goal here is to critically review the status of tRF research across sensory organs, including their primary sensory roles and secondary roles as exposed barrier tissues or environmentally exposed interfaces. The reviewed literature shows a conserved response of producing tRF as putative regulators across these organs. Specifically, it suggests that tRFs may participate in multiple biological processes, such as angiogenesis and immune regulation across sensory organs, which require rapid and efficient post-transcriptional regulation to maintain tissue homeostasis. However, the depth of evidence remains uneven, with diabetic conditions receiving most attention. The reviewed studies do not support unified models, in which the same tRFs regulate common targets across many sensory organs. Instead, the literature suggests that different tRFs sometimes converge on similar stress response pathways in different tissue contexts; however, in other cases, the involved tRFs and targets are different. For example, in eye-related diseases, several tRFs have been linked to retinal and choroidal neovascularization through the VEGF/HIF-1α endothelial pathway [18]. Related vascular and remodeling themes have also been described in skin, where tRFs are associated with Wnt/β-catenin and TGF-β/Smad pathways [68]. In the oral context, tRFs have been linked to microbial growth control and interferon-related responses [70]. 

Major limitations occur when uneven coverage is combined with research bias (in turn affected by funding bias), which takes several forms. One example is the focus on bacteria in the oral cavity, rather than purely on the function of the sensory organs present in that cavity. Further, certain organs have been long studied using a set of defined assays (e.g., Table 1), which frequently detect pre-determined phenotypes and related mechanisms. This further leads to bias towards known pathways that are already well studied, well annotated, and easier for primary authors to interpret. 

This limitation is especially important for type I studies, which often utilize simplistic approaches after sequencing small RNAs in different samples: to run software for differential tRF abundance and then for target prediction and Gene Ontology term classification. Such studies mostly neglect to provide further experimental evidence beyond a few qRT-PCR tests and ignore assumptions of the software tools used, often producing large and rather meaningless figures of functional enrichment or graphs of putative interactions without much thought or biological value. We also came across publications in which the authors claimed exorbitant and unrealistic numbers of gene targets (in one instance, >60,000—without regard to the fact that it is three times the human gene complement). Avoiding such cases, we reviewed and included type I papers primarily for the organs, in which detailed tRF studies were limited. Despite the lack of definitive evidence, type I papers often make claims about tRFs as potential biomarkers. Claims like these are difficult to refute, so we mentioned some of them here, but ultimately, leave it to the primary authors and readers to pass judgment on such statements.

While target prediction may be useful for generating hypotheses, it can further amplify preexisting knowledge bias because enrichment tools rely on databases that are themselves biased towards well-studied genes and pathways. Mechanistic interpretation is uneven across the reviewed studies, and some reports provide only predicted targets and pathway enrichment, whereas others include direct target validation or functional rescue experiments in disease-relevant models. Several methodological issues further restrict broad synthesis. Small RNA sequencing protocols differ between studies, and these differences can affect which specific tRFs are detected. tRNA modifications can interfere with reverse transcription, leading to under-detection or biased quantification of certain fragments. Inconsistent normalization methods, differences in read length selection, and incomplete validation of cleavage products further complicate cross-study comparison. Disease stage (including inflammatory level), treatment status, sample handling, and sequencing depth may also influence tRF abundance. Thus, we believe that the current literature supports a cautious synthesis view that tRFs are likely important stress-responsive regulators in sensory and environmentally exposed issues, but the apparent convergence on familiar pathways should be treated as hypothetical, rather than definitive. 

Extracellular vesicles (EVs) add an important layer to tRF studies. tRF and associated RNA binding proteins may be packaged into multivesicular bodies, released as exosomes, and taken up by recipient cells, where they may influence gene regulation and signaling [5]. EV-associated tRFs may provide a mechanism for cell-to-cell communication [5], which is very relevant within sensory microenvironments. This is especially important for sensory organs since these tissues contain interacting epithelial, immune, and microbial components [70]. For example, EVs derived from mesenchymal stem cells have been reported to carry tRFs that may protect retinal photoreceptors and modulate immune pathways [40]. In oral tissues, host-derived tRFs released in EVs may participate in host–microbe interactions by affecting bacterial growth. However, detection of a tRF in EVs or biofluids does not necessarily prove that it has a functional role. The latter will require stronger evidence, demonstrating selective loading of tRF cargo into EVs, their uptake by the recipient cell, a target engagement, and a measurable biological effect. Future studies should prioritize direct disease-relevant models, EV uptake, cargo function studies, and standardized sequencing, which will help determine whether the repeated appearance of shared pathways reflects truly conserved tRF biology.

## 6. Conclusions

Given the correlation observed between tRF expression levels and disease progression and clinical outcomes, more rigorous and well-controlled experimental studies are required to clarify the precise roles of tRFs and strengthen their translational relevance. tRFs represent an emerging and rapidly evolving field of research with significant potential to contribute to advances in multiple areas of disease biomedicine and precision medicine, provided that future studies adhere to stringent methodological and validation experiments. 

## Figures and Tables

**Table 1:** Common assays and models utilized to study eye-related tRFs.

Title	Common Assays/Models
**In vitro cell function assays**	Cell viability assay Cell proliferation assay (CCK-8, EdU on HUVEC-human umbilical vein endothelial cells) Protein binding (e.g., RNA pull-down assay) Blood vessel growth (e.g., matrigel plug assay); endothelial cell migration assay (scratch/wound healing or transwell migration) Inflammatory factor measurement (ELISA assay) Tube formation assay (branching and looping) Apoptosis assays (annexin V/PI, TUNEL)
**Vascular assays**	Vascular leakage assay (e.g., Evans blue assay) Acellular vessel assay (e.g., retinal trypsin digestion assay) Choroidal spouting assay Tip-cell formation assay
**In vivo models**	Laser-induced CNV (choroid neovascularization) mouse model Form-deprived myopia (FDM) model Oxygen-induced retinopathy (OIR) model
**Imaging analysis**	Fundus fluorescein angiography (FFA) Electroretinography (ERG) OCT (optical coherence tomography) Flat-mount choroidal staining (isolectin B4)

**Table 2:** Functions of skin.

Role	Specific Function	Description
**Protection and Barrier**	Mechanical & Chemical Defense	Protects internal organs, muscles, and tissues from physical trauma, friction, and external chemicals.
**Protection and Barrier**	Water Regulation	A semi-impermeable barrier to prevent the body from losing excessive fluids (dehydration) and absorbing too much water from the environment.
**Protection and Barrier**	UV Shielding	Contains melanocytes that produce melanin, deflecting harmful ultraviolet (UV) radiation from the sun to prevent cellular DNA damage.
**Immune Defense**	First Line Defense	Serves as the initial physical barrier against invading pathogens, bacteria, and viruses.
**Immune Defense**	Chemical Secretions	Secretes antimicrobial peptides and mildly acidic sweat that kill bacteria and fungi on its surface.
**Immune Defense**	Cellular Response	Utilizes specialized immune cells (like Langerhans cells) to detect foreign invaders and trigger the body’s adaptive immune system.
**Temperature** **Regulation**	Cooling	Releases perspiration from sweat glands which cools the body as it evaporates; dilates (widens) blood vessels to release internal heat.
**Temperature** **Regulation**	Warming	Constricts (narrows) blood vessels to keep warm blood near vital organs; utilizes fat in the hypodermis layer as thermal insulation.
**Sensation**	Sensory Perception	Uses a vast network of nerve endings and receptors to perceive touch, pressure, vibration, heat, cold, and pain, allowing interaction with the environment and injury avoidance.
**Synthesis and Storage**	Vitamin D Production	Synthesizes Vitamin D within specialized cells when exposed to sunlight, which is essential for bone health and calcium absorption.
**Synthesis and Storage**	Reservoir	Acts as a metabolic storage center for water, lipids (fats), and blood.
**Excretion**	Waste Elimination	Eliminates small amounts of waste products (urea, ammonia, and excess salts) through the production of sweat and sebum, acting as a secondary excretory system to the kidneys.

**Table 3:** Number of tRFs associated with various oral and pharyngeal diseases.

Number of tRFs Studied	Diseases/Conditions	Related-Area/Organ
2	Microbiome modulation	Oral
15	Cancer (oral squamous cell carcinoma, laryngeal carcinoma, hypopharyngeal carcinoma, oral melanoma, esophageal carcinoma)	Oral
2	Non-syndromic cleft palate	Oral
8	Viral diseases (RSV, SARS-CoV-2)	Nasopharyngeal
2	Respiratory tract inflammation (asthma)	Nasopharyngeal
1	*Candida auris* infection	Ear

## Data Availability

No new data were created or analyzed in this study. Data sharing is not applicable to this article.

## Appendix A

**Table A1:** tRF sequences and their corresponding IDs and references. Sequences were extracted from the paper text whenever possible and otherwise by a good-faith effort from Figures and Supplementary Materials (in some papers, nothing was found). Sequences are colored by the corresponding paper type (Type I: green; Type II: purple; and Type III: blue).

Sequence	ID	Reference
TTTTATTCTCGTGGGCGAAG	T12a	[12]
GCAAGAGACCGTGCA	T13a	[13]
CGCAGGTCTTGTAAACCAG	T16a	[16]
TCCCTGGTGGTCTAGTGGTTAGGATTCGGCG	T18a	[18]
GCATGGGTGGTTCAGT	T19a	[19]
GCCACCAAGATCGGAAGAGC	T20a	[20]
TCGGAGGCTTTGTTTT	T26a	[26]
GTTTCCGTAGTGTAGTGGTTATCACGTTCGCCT	T27a	[27]
AGTTGGGAGAGCGTTAGACTGAAGATCA	T28a	[28]
ATCCCACCGCTGCCACCA	T29a	[29]
GCCGTGATCGTATAGTGGTTAGTACTCTGCGTTGT	T30a	[30]
GGGTCAAATCTCGGTGGAAC	T31a	[31]
AGAGTGGCGCAGCGGAAGC	T32a	[32]
CGATCTAGTCAAATGCTCTACCAC	T32b	[32]
GCCGCTGGTGTAGTGGTATC	T32c	[32]
CGTTTCTGTAGTGTAGTGGTTATC	T32d	[32]
GCTCTGTGGCGCAATGGATAG	T32e	[32]
ACAGTCCGACGATCTCCCTG	T32f	[32]
TAGGTCGCTGGTTCGTTTC	T33a	[33]
TCCGACGATCTCCCCGTAC	T34a	[34]
CGACGATCTCCCTGTTCGG	T34b	[34]
CCGACGATCAAGAAAGATTGC	T34c	[34]
AGTCCGACGATCAATTGTATCC	T34d	[34]
GAAGCGGGTGCTTCACTTTT	T35a	[35]
GCCGAGATCGTATAGTGGTTAGTACTCTGCA	T37a	[37]
GCATGGGTGGTTCAGTGGTAGAATTCTCGC	T37b	[37]
GCATTGGTAGTTCAATGGTAGAATTCTCGCC	T37c	[37]
GCGCCGCTGGTGTAGTGGTATCATGCAAGATT	T37d	[37]
AGCAGAGTGGCGCAGCGGAAGCGTGCTGGGC	T37e	[37]
GCCGTGATCGTATAGTGGTTAGTA	T38a	[38]
CGAACCGAGGTTGCTGCGGCC	T39a	[39]
ATCCGAGTCACGGCACCA	T42a	[42]
GCGCCGCTGGTGTAGTGGTATCATGCAA	T45a	[45]
GGTTCCATAGTGTAGTGGTTATCACGTCTGCT	T46a	[46]
GCATTGGTGGTTCAGT	T46b	[46]
GTTTCCGTAGTGTAGTGGTTATCACGTTCGCC	T46c	[46]
TCGAATCCCACTCCTGACACC	T47a	[47]
CTAAGCCAGGGATTGTGGGT	T47b	[47]
ACCCCACCTCTGGTACCA	T55a	[55]
GCCGTGATCGTATAGTGGTTAGTACTCT	T57a	[57]
GCGCCGCTGGTGTAGTGGTATCATGCAA	T59a	[59]
TCCCTGGTGGTCTAGTGGTTAGGATTCGG	T59b	[59]
GTTTCCGTAGTGTAGTGGTCATCACGTTCGCC	T59c	[59]
AGGCTCGTTGGTCTAGGGGTA	T60a	[60]
TGACTGGACCTTTCTTTTGC	T61a	[61]
TCGAATCCGGCTCGAAGGACCA	T61b	[61]
GCCCCAGTGGAACCACCAAG	T61c	[61]
GTTGGTGGTATAGTGGTAAG	T61d	[61]
CTCTGTGGCGCAATGGACGA	T61e	[61]
GTGTAAGCAGGTTCGGTTCC	T61f	[61]
GCCGTGATCGTATAGTGGTTAGTACTCTGCGTTG	T62a	[62]
TCCCACATGGTCTAGCGGTTAGGATTCCTGGTTT	T63a	[63]
GCGCCGCTGGTGTAGTGGTATCATGCAAGA	T64a	[64]
GAGAAAGACATAATGGTTATGAC	T65a	[65]
GAAAAAGTACTGCAAGAACT	T65b	[65]
AGGAGTTTAGGTTAGACCAGACC	T65c	[65]
GTAGTCGTGGCCGA	T66a	[66]
GGGGGTATAGCTCAGGGGTAGA	T67a	[67]
GGGGGTATAGCTCAGTGGTAGAG	T67b	[67]
GGGGGTGTAGCTCAGTGGTAGAG	T67c	[67]
GCATGGGTGGTTCAGTGGTAGAATTCTCG	T68a	[68]
CCGGCTAGCTCAGTCGGTAGAGCATGAGA	T69,70a	[69,70]
GGGGGTATAGCTCAGTGGGTAGAGCAT	T69,70b	[69,70]
GTCTCTGTGGCGCAATGGAC	T71a	[71]
ATTGGTCGTGGTTGTA	T71b	[71]
TCCGGCTCGAAGGACC	T71c	[71]
GCGCCGCTGGTGTAGTGGTATCATGCAAGATTC	T75a	[75]
GTTCCGGCTAGCTCAGTCGTT	T76a	[76]
AGGAGTTTAGGTTAGACCAGACC	T77a	[77]
GCATGGGTGGTTCAGTGGTAGAATTCTCGCC	T78a	[78]
GCGTTGGTGGTATAGTGGTAAGCATAGCTGCCTTC	T79a	[79]
GGGGGTATAGCTTAGCGGTAGAGCATTTGACTG	T79b	[79]
GCTTCTGTAGTGTAGTGGTTATCACGTT	T80a	[80]
GCTGTGATGGCCGAGTGGTTAAGG	T80b	[80]
TCGACTCCTGGCTGGCTCGCCA	T80c	[80]
AACGTGATAACCACTACACTACAGAAGC	T81a	[81]
TCCCTGGTGGTCTAGTGGTTAGGATTCGGCG	T82,83a	[82,83]
TCCCTGGTGGTCTAGTGGTTAGGATTCGGCG	T84a	[84]
TCCCTGGTGGTCTAGTGGTTAGGATTCGGC	T85a	[85]
TCCCTGGTGGTCTAGTGGTTAGGATTCGGCGC	T87a	[87]
GCATTGGTGGTTCAGTGGTAGAATTCTCGC	T87b	[87]
TGAATCCATCAAGTTACCAATCTTGTCTACGGAGTATA	T88a	[88]
GAGGGCCACCGGTTCGACCGATTCGCGCTCTGACATTACACTAACATTA	T88b	[88]
